# Vimentin Intermediate Filament Rings Deform the Nucleus During the First Steps of Adhesion

**DOI:** 10.3389/fcell.2019.00106

**Published:** 2019-06-17

**Authors:** Emmanuel Terriac, Susanne Schütz, Franziska Lautenschläger

**Affiliations:** ^1^Leibniz Institute for New Materials, Saarbrücken, Germany; ^2^Faculty of Natural Sciences and Technology, Saarland University, Saarbrücken, Germany

**Keywords:** vimentin, adhesion, nuclear deformation, ring, cell spreading

## Abstract

During cell spreading, cells undergo many changes to their architecture and their mechanical properties. Vimentin, as an integral part of the cell architecture, and its mechanical stability must adapt to the new state of the cell. This study focuses on the structures formed by vimentin during the first steps of cell adhesion. Very early, ball-like structures, or “knots,” are seen and often vimentin filaments emerge in the shape of rings around the nucleus. Although intermediate filaments are not known to be associated to motor proteins to form contractile systems, these rings can nonetheless strongly deform the cell nucleus. In the first 6 to 12 h of adhesion, these vimentin knots and rings disappear, and the intermediate filament network returns to the state seen before detachment of the cells. As these vimentin structures are very transient in the early steps of cell spreading, they have rarely been described in the literature. However, they can also be seen during mitosis, which is an event that involves partial detachment and re-spreading of the cells. Interestingly, the turnover dynamics of vimentin are reduced in both the knots and rings, compared to vimentin in the lamellipodia. It remains to define how the force is transmitted from the ball-like structures to the rings, and to measure the impact of such strong nuclear deformation on gene expression during cell re-spreading and the rearrangement of the vimentin network.

## Introduction

During embryogenesis, as also in the more detrimental context of metastasis, cells translocate from their original surrounding or tissue in other tissues (Greenburg and Hay, 1982; Hay, 1995; Davies, 1996; Nieto et al., 2016; Li et al., 2017; Roche, 2018). Upon arrival at their new location, the cells need to anchor to their new environment. During these processes, to correctly migrate, cells detach, by at least partial down-regulation of the expression of E-cadherin, among other factors, and up-regulation of the expression of mesenchymal markers, like N-cadherin and vimentin (Grunert et al., 2003; Christofori, 2006). This is referred to as epithelial-to-mesenchymal transition (Kalluri and Weinberg, 2009; Lamouille et al., 2014). Eventually, the cells will re-spread in a new environment, and will thus undergo the reversed transition called mesenchymal-to-epithelial transition (Davies, 1996). *In vitro* cell migration studies often require full detachment of cultured cells from their original surface, with these studies then carried out once the cells have re-adhered a new surface. To understand how cells move from one surface, or tissue, to another, an understanding of the mechanisms of cell detachment and cell spreading is crucial.

Several studies have compared cells in adhered and suspended states, and it is clear that the cellular properties are very different. In particular, the mechanical properties of the cells are strongly altered in those two cases (Maloney et al., 2010; Chan et al., 2015). These detachment and spreading transitions whereby cells can adapt to a new state have been studied for many decades (Aoyama and Okada, 1977; Urushihara et al., 1977; Okano et al., 1995; Wakatsuki et al., 2003; Lynch et al., 2013).

Cellular mechanics are mainly governed by the cytoskeleton (Fletcher and Mullins, 2010) through the cytoskeleton fiber types: actin microfilaments, microtubules, and intermediate filaments. Several proteins fall in this last category (Fuchs and Weber, 1994; Herrmann and Aebi, 2016) and it has been shown that different cell types express different intermediate filament proteins, and that failure of correct expression of intermediate filament proteins can lead to several diseases (Danielsson et al., 2018). In the context of epithelial-to-mesenchymal transition, and in the reverse process, the expression for intermediate filaments changes between mainly keratin (epithelial) to mainly vimentin (mesenchymal).

In the present study, we show that during the first hours of adhesion, vimentin forms ball-like structures in close vicinity to the nuclei. These “knot” structures are transient and generally disappear within the first 6 h after re-spreading of the cells. These structures are often associated with vimentin rings around the nuclei, which can also slide along the nuclei. However, most surprisingly, while the non-polar vimentin filaments are not associated with molecular motors, these rings can exert force on the nuclei, and can even cause them to become deformed. We further show that this transient phenomenon can also be observed after re-spreading of mitotic cells.

## Materials and Methods

### Cell Culture

Immortalized retinal pigmented epithelium (hTERT-RPE1) cells were cultured in DMEM/F12 medium (Gibco) with 10% fetal bovine serum (Fisher Scientific), 1% GlutaMAX (Fisher Scientific) and 1% penycilin/streptomycin (Gibco). Human foreskin fibroblasts (HFFs) were cultured with DMEM (Gibco) supplemented in the same manner.

Wild-type hTERT-RPE1 cells were from American Type Culture Collection (ATCC CRL-400). TALEN-edited cell lines included hTERT-RPE1 cells expressing mEmerald-vimentin and mTagRFP-tubulin, and HFFs expressing mEmerald-vimentin, and these were kindly provided by Gaudenz Danuser (UT Southwestern, Dallas, TX, United States). The genome editing has been described previously (Gan et al., 2016; Costigliola et al., 2017). These cell lines express the aforementioned proteins at levels comparable to their respective parental cell lines. The amount of the fluorescent protein, compared to the total amount of protein, was reported to be 4% for mEmerald-vimentin in HFFs, 8 and 5% for mEmerald-vimentin and mTagRFP-tubulin, respectively, in hTERT-RPE1 cells.

### Immunostaining

Cells were fixed for 10 min in 4% paraformaldehyde in phosphate-buffered saline (PBS) and were rinsed three times for 5 min each in PBS. The cell membranes were permeabilized for 10 min in a 0.5% Triton X-100 in PBS, followed by three rinsing steps. Before immunostaining, the cells were blocked in 3% bovine serum albumin in PBS solution for 1 h. Vimentin was stained overnight with an Alexa Fluor 647 tagged human anti-vimentin V9 antibody (sc-6260; Santa Cruz) at 0.2 μg/mL in 3% bovine serum albumin in PBS. Actin was stained with fluorescently labeled phalloidin (phalloidin-iFluor 488; ab176753; Abcam) at a dilution of 1:1000, accordingly to the manufacturer protocol. After staining, the cells were rinsed three times in PBS and once in MilliQ water, and then they were mounted on glass slides using Fluoromount-G (Thermo Fisher Scientific), which contained DAPI to counterstain the cell nuclei.

### Imaging

Epi-fluorescence images were acquired on different inverted microscopes (Ti-Eclipse; Nikon). The light sources used were either an Intensilight Epi-Fluorescence illuminator (Nikon) or a Sola Light engine (Lumencor). The microscopes were equipped with a temperature controlled (37°C) environmental chamber (Okolab) that provided 5% CO_2_ and 100% humidity for the live-cell imaging. Confocal images were acquired on a confocal microscope (LSM 880; Zeiss). For live-cell imaging, the confocal images were acquired on an inverted microscope (Ti-Eclipse; Nikon) equipped with a Yokogawa spinning disk head (CSU-W1; Andor Technology) and a “fluorescence recovery after photobleaching/ photoactivation” (FRAPPA; Andor Technology) module. Live-cell imaging was performed in 23 mm diameter glass-bottomed dishes (World Precision Instruments). When mentioned, the dishes were pre-coated after 30 s of activation with plasma (Harrick PDC 32G), with a 25 μg/mL solution of bovine plasma fibronectin (F1141; Merck) and rinsed three times with PBS before adding cells.

## Results

### Vimentin Ball-Like Structures and Rings Deform the Cell Nuclei During the First Hours of Adhesion

To investigate the cytoskeleton of single cells during their spreading, we used epithelial-like hTERT-RPE1 cells that were genetically edited to express mEmerald-vimentin and mTagRFP-tubulin (Gan et al., 2016). RPE cells are of epithelial origin, although in culture they show attributes of both epithelial cells, such as collective migration in wound-closure assays (Gan et al., 2016), and mesenchymal cells, such as high levels of vimentin expression (Yang et al., 2018). Of note, they also show higher expression of N-cadherin than E-cadherin (Youn et al., 2006), which is also a marker attributed to mesenchymal phenotypes. As such, RPE cells are a good model for initiation of mesenchymal-to-epthelial transition, i.e., for cell attachment and spreading to a new surface.

**FIGURE 1:**
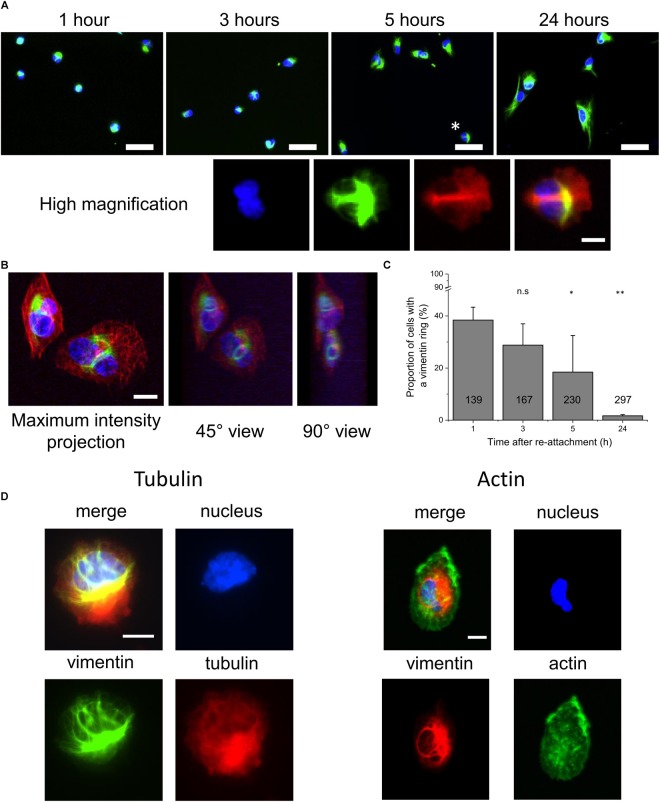
Appearance of vimentin rings around the nuclei during the first hours of cell adhesion and spreading. **(A)** Top: Representative fluorescence images of fixed RPE1 cells after 1, 3, 5, and 24 h of adhesion on medium-incubated glass slides (20× magnification). Bottom: Magnification of the cell indicated with a ^∗^ above. Green, vimentin; blue, nucleus. Scale bar: 50 μm (top); 10 μm (bottom) **(B)** Representative spinning disk confocal images of a vimentin ring. Left: maximum intensity projection in height image. Middle, right: three-dimension representation of left image at 45° and 90° angles, respectively. Green, Vimentin; red, tubulin; blue, nucleus. Scale bar: 10 μm. **(C)** Quantification of the proportion of cells with vimentin rings, based on fluorescence images of **(A)**. Data are means ± standard deviation; the number in each column corresponds to the total number of cells analyzed; ^∗^ and ^∗∗^*p* < 0.05 and *p* < 0.01, respectively, compared to the 1 h condition, one-way ANOVA with Bonferonni’s test. **(D)** Representative images of high magnification (100×) fluorescence images of cells after 3 h of adhesion and spreading. Left: Green, vimentin; red, tubulin; blue, nucleus. Right: Red, vimentin; Green, actin; blue, nucleus. Scale bars: 10 μm.

These cells were allowed to spread on glass coverslips, which were incubated overnight in the cell growth medium. They were then fixed at different times after re-adhesion (1, 3, 5, and 24 h). The nuclear staining was added prior to the epi-fluorescence imaging. Figure 1A shows representative images of the cells at these times. In these images, large ball-like structures, or knots, can be seen close to the nuclei. In some cases, those structures were accompanied by thin lines of vimentin that span the nuclei, and which were related to nuclear deformation. As an example, Figure 1A (zoom) shows a high-magnification image of a cell.

To determine whether these linear structures corresponded to a more complex three-dimensional (3D) assembly, confocal images were obtained (Figure 1B). Through to the projections of the 3D images reconstructed at different angles, it could be seen that the thin vimentin lines previously observed corresponded to rings of vimentin around the nucleus. These rings appeared to be under enough tension for local deformation of the nuclei.

We quantified the numbers of cells that had vimentinn rings at 1, 3, 5, and 24 h after re-adhesion (Figure 1C). Here, we noted that some cells had several rings, and some of these rings had more of a shape of a shaft around the nuclei, i.e., thick bundles of vimentin filaments, seen in the high-magnification shown in Figure 1A, rather than as thin structures. The number of cells with a vimentin ring rapidly decreased during the first hours of spreading, from nearly 40% after 1 h, to around 20% after 5 h (Figure 1C). These structures are very rarely seen after 24 h of cell adhesion.

To determine whether these effects were a consequence of the fluorescent tag fused to vimentin and tubulin, we performed immunofluorescence analysis in wild-type RPE1 cells. Here there were also rings, and therefore we concluded that the rings were not artifacts of the genetic editing performed on these cells (Supplementary Figure S1). In order to assess the need of vimentin to form the ring structures, we compared cells during adhesion after they were treated for 30 min in suspension with withaferin A, a steroidal lactone that has been shown to destabilize the vimentin filaments network, and is not expected to be lethal at short time scales (Grin et al., 2012). We observed that rings were appearing at a later time in the case of the withaferin A treated cells. We could also see that the appearance of the rings were corresponding to synchronous nuclear deformation (Supplementary Materials and Methods, Supplementary Figure S2, and Supplementary Movies S1, S2).

As vimentin intermediate filaments are known to interact with other cytoskeletal components, such as actin (Wiche et al., 1993; Cary et al., 1994; Foisner et al., 1995; Svitkina et al., 1996; Esue et al., 2006) and tubulin (Svitkina et al., 1996; Sakamoto et al., 2013; Gan et al., 2016), we looked for these two proteins in these rings. Figure 1D shows representative images of cells with vimentin rings where they were also stained for actin and tubulin. While the rings were seen in the vimentin channels and the nuclei are deformed, there does not appear to be any link with actin. For tubulin, its association with the ring was possible, as seen in the high-magnification panel of Figure 1A, but not necessary as seen in Figure 1D.

Altogether, these data show that during the first hours of cell adhesion, vimentin intermediate filaments form ball-like structures in close vicinity to the nuclei. Thin vimentin filaments then emerge from these vimentin “knots,” and they appear to form rings around the nuclei. These rings appear to deform the nuclei and to create invaginations in the nuclear surface. They also generally appear not to be related to other cytoskeletal structures, as actin is not found in them, and as tubulin is not necessarily present.

### Rings Are Transient Structures That Slide Along the Nucleus

**FIGURE 2:**
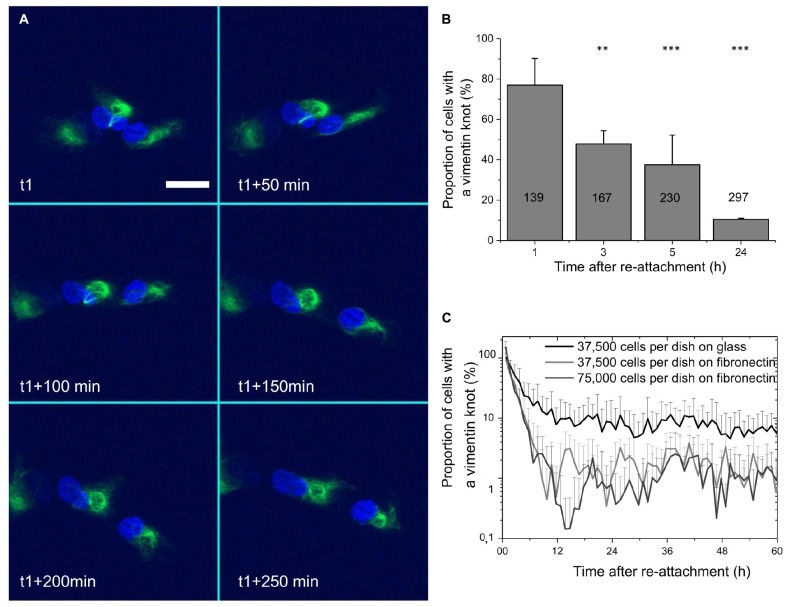
Vimentin rings are transient and are accompanied by vimentin knots. **(A)** Representative images of deformation of a nucleus by a vimentin ring and its recovery after the sliding of the vimentin ring. Spinning disk confocal images were acquired at different times after call adhesion (t1, 400 min). Green, vimentin; blue, nucleus. Scale bar: 20 μm. The full movie is available as Supplementary Material. **(B)** Quantification of the proportions of cells with vimentin ball-like structures during cell adhesion and spreading, based on quantification of Figure 1B; the number in each column corresponds to the total number of cells analyzed; ^∗∗^ and ^∗∗∗^*p* < 0.01 and *p* < 0.001, respectively, compared to the 1 h condition, one-way ANOVA with Bonferroni’s test. **(C)** Quantification of proportions of cells with vimentin ball-like structures, over 60 h of cell adhesion and spreading, including different coatings and different cell concentrations. Each time point represents the proportion of knot for a minimum of 125 cells for the 37,500 cells per dish conditions and 250 cells for the 75,00 cells per dish condition.

As the ball-like structures and rings disappear with time, to better define their dynamics, we carried out time-lapse recordings over several hours of cell spreading. For the rings, it was difficult to establish the timing of their disappearance, especially when several of them could be present within a single cell. Figure 2A (extracted from Supplementary Movie S3) shows an example of the disappearance of a ring. In this case, the ring started to disappear about 5 h after cell adhesion. At that moment, it slid along the nucleus. Upon reaching the edge of the nucleus, the ring became smaller, and then it fused into the knot from which it had initially emerged. We could also see that the deformation of the nucleus was aligned with the position of the vimentin ring. In the Supplementary Figure S3, images of the same sequence than Figure 2A are depicted with a smaller time interval and with black and white balance settings chosen in order to saturate the vimentin knot signal to better emphasize the vimentin rings. Arrows are aligned to show that the deformation of the nucleus corresponds to the position of a vimentin ring.

This raised the question of the stability of the knot itself. To quantify this, the images used in the quantification of Figure 1B were further analyzed, to determine the proportions of ball-like structures at the four different times. Considering the proportions of cells with a vimentin ring (Figure 1B), Figure 2B shows a similar decrease in the proportions of these ball-like vimentin knots, compared to the vimentin rings. However, the total proportions for the knots are a lot higher. Indeed, after 1 h of spreading, more than 75% of the cells showed the knot structures. This decreased to roughly 10% after 24 h.

To better quantify the disappearance of the vimentin rings and the vimentin ball-like structures during cell re-attachment, the spreading of the cells was monitored over 60 h. Time-lapse movies were analyzed automatically with FiJi software (Schindelin et al., 2012) by setting a threshold that excluded vimentin as the more diffused network (low homogeneous intensities taken from the last time points), and thus quantified only the (brighter) ball-like structures. In parallel, the numbers of cell nuclei were automatically quantified (according to DAPI staining). In Figure 2C, the proportions of the ball-like structures over 60 h are shown.

At the same time, the potential influences of cell-cell adhesion, cell secretion, and cell-substrate affinity were tested, in terms of varying the concentrations of the cells added to the dishes (either 37,500 or 75,000 cells per 23 mm diameter dish) or the coatings of the dish surface for cell attachment (bare glass, or 25 μg/mL fibronectin pre-coating).

Here, there were strong decreases in the proportions of cells showing the ball-like structures during the first hours of cell spreading. This stabilized between 2 and 10% after 12 h. Over this time, while the number of cells added to the dishes appeared to have little effect, the pre-coating of the dish had an important role. With the uncoated glass dishes, the proportion of cells with vimentin knot structures stabilized to 10% after 12 h, where it then remained for the duration of the experiment. For the fibronectin-coated dishes, the proportion of cells with vimentin knot structures stabilized to the lower level of around 2% after 12 h, and then remained low throughout.

To determine whether these changes in the appearance of vimentin rings and the ball-like vimentin knots are a particular phenotype of the epithelial-like hTERT-RPE1 cell type, this was repeated in HFFs, that were genetically engineered in the same manner as the RPE cells, although only regarding for vimentin (Costigliola et al., 2017). Similar data were obtained (Supplementary Figure S4), which thus showed that this phenotype is not cell-type specific.

These data thus demonstrate that during cell attachment and spreading, while a relatively low proportion of cells show the nucleus-deforming rings of vimentin, the majority show the ball-like juxtanuclear vimentin knots. These two structures are not fully independent of each other, as during the sliding of a ring along the nucleus, the ring remains anchored to the ball-like structure it emerged from. Also, these ball-like structures only persist through the first few hours of cell adhesion and spreading, and after roughly 12 h, very few cells still show them. Furthermore, this behavior is not a phenotype specific being to the RPE cells, and the strength of the adhesion of the cells to different surfaces appears to have a role in the rate of disappearance of these ball-like vimentin knots.

### Vimentin Ball-Like Structures and Rings Are Also Observed During Mitosis

**FIGURE 3:**
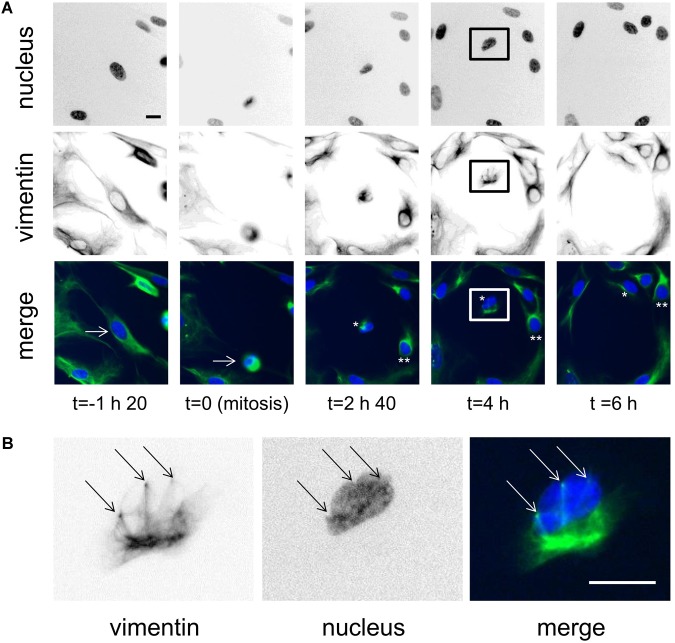
Vimentin knots and rings are observed during mitosis. **(A)** Representative images of a cell dividing over time. Initially, the cell is shown 1 h 40 before cell division, then at cell division, and at three times after cell division. The original cell is indicated by the arrow on the two first panels. The daughter cells are indicated as ^∗^ and ^∗∗^ in the color-merged views. Scale bar: 20 μm. **(B)** Magnification of the daughter cell [framed in (A)] showing the vimentin rings, (arrows). Scale bar: 10 μm.

During mitosis, cells do not fully detach, but they do ‘round up’ for the cell division, and during cytokinesis, both of the daughter cells spread back onto the surface. Here, during this re-spreading of the daughter cells after division, it was possible to see the ball-like vimentin knots, and sometimes also the nucleus-deforming vimentin rings (Figure 3 and Supplementary Movie S4). However, these were not always clear in all cells, nor did they necessarily proceed in parallel. Indeed, Figure 3A, shows a case of daughter-cell asymmetry over the first 6 h from mitosis. In one daughter-cell, from 2 h 40 min from mitosis (Figure 3A, ^∗∗^), the vimentin network was rapidly reformed without any vimentin rings seen, and thus without deforming the nucleus. On the other hand, the second daughter cell, (Figure 3A, ^∗^, see also higher magnification in Figure 3B) showed a vimentin knot structure that was accompanied by nucleus-deforming vimentin rings while it was spreading (4 h after mitosis). This nucleus was seen to be bound by three nucleus-deforming vimentin rings (Figure 3B). In this case, the ball-like vimentin knot remained for longer than for the other daughter cell. This thus also shows that the vimentin ball-like knot or the deformation of the nucleus by vimentin rings might delay the reassembly of the vimentin network after cell division.

### Vimentin Turnover Is Lower in the Ring Than in the Ball-Like Structure

**FIGURE 4:**
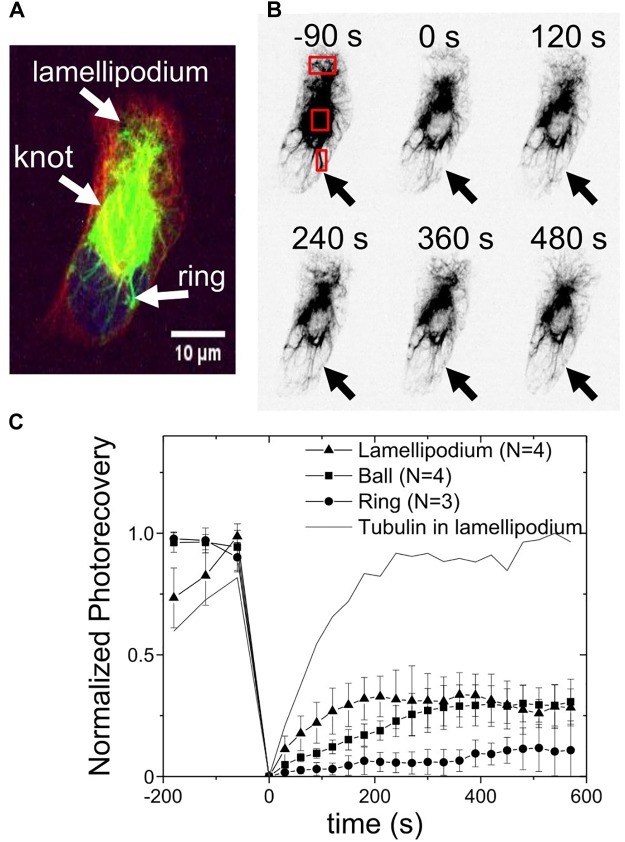
Vimentin dynamics in the knot and ring structures measured by fluorescence recovery after photobleaching. **(A)** Representative image of a cell before photobleaching and the labeling of the three regions of interest: lamellipodium, and vimentin knot and ring. Green, vimentin; red, tubulin; blue, nucleus. **(B)** Fluorescence recovery after photobleaching time-lapse. Red squares: Three regions where photobleaching occurred, and where recovery was measured. Arrows, where the ring was initially. **(C)** Photorecovery curves of vimentin in the lamellipodium (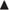), knot (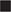), and ring (•). Line, example of recovery of tubulin in the lamellipodium (same region), as comparison.

The dynamics of the vimentin in the ball-like knot and the ring structures, were examined using fluorescence recovery after photobleaching (FRAP) experiments. Initially, the vimentin was bleached in three different regions: the lamellipodium and the juxtanuclear ball and ring (Figure 4A,B). Using a high intensity laser for the bleaching phase also allowed monitoring of the recovery of tubulin in the lamellipodium, as a control for recovery. These data were analyzed according to the protocol of Kappel and Eils (Kappel and Eils, 2004) and plotted accordingly (Figure 4C). Due to the low number of cases and the lack of a more refined model for the dynamic of vimentin, only the final values of recovery (plateau values at 10 min) were compared.

A recovery of 30% to 35% of the vimentin bleaching in the vimentin ball-like knots and the lamellipodia was seen over the 600 s recording following the bleaching. As a control, the recovery of the bleaching of the microtubules was measured in the same lamellipodial region, which reached 100% over the time of recording. However, only about 10% of the vimentin in the rings was recovered within the same time. At this level of analysis, the vimentin in the knots and lamellipodia appears to be 3–5 fold more dynamic than the vimentin in the ring structures.

## Discussion

During the spreading of cells in a new tissue *in vivo* or in a new sample environment *in vitro*, they undergo drastic changes in their architecture, protein expression, and mechanical properties. Cells can adapt to such transitions due to their plasticity. Here, we have looked at the rearrangement of the vimentin intermediate filaments network during cell attachment and spreading as this protein is one contributor to cell mechanics (Wang and Stamenovic, 2000; Mendez et al., 2014).

Prahlad et al. (1998) described the interplay between vimentin filament precursors and microtubules at the cell periphery during the early stages of cell spreading. Furthermore, Lynch et al. (2013) showed that this interaction between vimentin and microtubules was required for spreading of the endoplasm, whereby for correct spreading of the endoplasmic cage, small fragments of vimentin are connected following their transport along microtubules. In both of these studies, the images presented showed a large accumulation of vimentin around the nuclei.

In the present study, we focused on these ball-like structures, or “knots,” of vimentin. We detected vimentin rings that spanned from this structure, using confocal microscopy. While no motors are known to act directly on vimentin filaments to provide a contractile system, our data show that these rings can cause deformation of the nuclei of the spreading cells.

These rings are reminiscent of the structures reported by Parysek and Eckert (1984) for spreading neutrophils. They described loose juxtanuclear knots of vimentin from which filaments can radiate. However, they did not further investigate the radiating filaments, which might have also included rings of vimentin. Furthermore, while electron micrographs of spreading cells have shown deformed nuclei, it would have been difficult to attribute this effect specifically to the vimentin rings, particularly as neutrophil nuclei are known to be poly-lobulated (Campbell et al., 1995).

Ring-like structures composed of intermediate filaments and their creation of nuclear invaginations were previously reported by Kamei (1994) in different cancer cell lines, where they were mainly prominent in PaCa cells (i.e., undifferentiated human pancreatic carcinoma cells). However, neither the resilience of the structures nor their temporal relationships to cell adhesion and spreading were touched upon, as the main aspects that we have addressed in the present study. However, this PaCa cell line might represent a specific model for the formation of long lasting vimentin ring structures.

During the spreading of the cells in the present study, the two vimentin structures, as knots and rings, disappeared over time. After 3 h, less than 50% of the cells still showed those structures, and after 12 h, they were almost all gone, for both the hTERT-RPE1 cells and HFFs during cell attachment and spreading on fibronectin-coated glass. This might be the reason why these vimentin ring structures have not been described previously. Indeed, most *in vitro* studies have been actually performed on cells that have been left to adhere for several hours, to give the cells time to adapt to their new environment.

A reversed effect was reported by Hirano et al. (1992). They treated 3T3 fibroblasts with the protein phosphatase inhibitor calyculin A. This led to partial detachment of the cells with the description of ball structures of vimentin that formed around the nuclei and deformed them. In some of their micrographs some vimentin ring-like structures can indeed be recognized. However, their study might be more difficult to interpret, as calyculin A is often use as a vimentin disassembling molecule (Eriksson et al., 2004).

Interestingly, it is possible to see ball-like vimentin structures, and even vimentin rings, in cell that have undergone mitosis. During cell division, the cells partially detach from their substrate to assemble the mitotic spindle. Once mitosis has occurred, then they re-spread on the surrounding surface. During mitosis, vimentin interacts with the actin cortex via its tail domain, and indeed, selective deletion of amino acids in the tail domain of vimentin has shown to impair cell progression through mitosis (Duarte et al., 2018).

Here, in a daughter cell, we observed the assembly of several vimentin rings that resulted in the deformation of the cell nucleus, which was not seen for the other daughter cell. It has recently been shown that vimentin is involved in asymmetric partitioning of “Juxta nuclear quality control” (JUNQ) inclusion bodies, which are responsible for the degradation of misfolded proteins (Ogrodnik et al., 2014). Our observations are in line with what they reported, although we also show here that similar behavior can be seen outside of the mitosis context.

We also quantified the turn-over of vimentin in these ball-like vimentin knots and rings, and in lamellipodia, using FRAP. Compared to other cytoskeletal fibers, like microtubules, the vimentin filaments are much less dynamic. This is in line with previous reports (Yoon et al., 1998). It is also especially true for formed filaments, which have lower rates of subunit exchange than unit-length filaments (Robert et al., 2015). In the present study, we even show differences in the recovery amount of vimentin along the rings, in the vimentin knots and in the lamellipodia.

It can be hypothesized that the vimentin rings interact with the nuclei, and hence this might slow down the exchange of the vimentin. It has been shown, that nesprin-3 connects both plectin and vimentin to the nuclear envelope in Sertoli cells (Ketema et al., 2013). In the ball-like vimentin structure, the vimentin turn-over also appear to be impaired compared to the lamellipodia as the signal needs more time to reach the final recovery value. These reduced vimentin dynamics might also arise from interactions between vimentin and other cellular proteins: indeed inactive ROCK might represent one such factor (Sin et al., 1998). This interaction has been suggested to be a way for migrating fibroblast to maintain their polarity, by controlling both the stress at the back of the cell and the Rac1-activating vimentin squiggles at the front of the cell (Terriac et al., 2017). This is also not necessarily the only occurrence of interactions between intermediate filaments and the contractile system of cells; another study has indeed shown that keratin K8/K18 can modulate cell stiffness through its interactions with the RhoA-ROCK pathway (Bordeleau et al., 2012).

We hypothesize that interactions between ROCK and vimentin are involved in the observed structures here. During the early stage of cell spreading, cells first down-regulate active ROCK via phosphorylation of p190RhoGAP (Arthur et al., 2000; Huveneers and Danen, 2009). This step is assumed to allow the relief of the stress in the spreading cell. Later on, ROCK becomes active through activation of p190RhoGEF, and can then be used for stress fiber formation, as well as for polarity initiation (Lim et al., 2008). Vimentin, on the other hand, can be phosphorylated at the amino-acid S71 by ROCK, which leads to its own reorganization (Lei et al., 2013; Hyder et al., 2015). We speculate that in our case the disappearance of the vimentin ball-like structures during cell adhesion is mainly due to the activation of ROCK over time. Moreover, integrin signaling is involved in the activation of RhoA during the later stage of cell adhesion (Danen et al., 2002). This is in line with the present study, where we show that on uncoated glass surfaces, the ball-like vimentin knots required more time to disappear.

## Conclusion

In conclusion, our study shows that during cell adhesion and spreading, vimentin intermediate filaments can assemble into ball-like structures, or knots. These vimentin knots may be reminiscent of the previously vimentin assembled network before cell detachment. During the re-spreading of cells on a new surface, the vimentin knots might be the origin of the vimentin rings that are seen to strongly deform the cell nuclei. The origin of the force that must act on the nuclei during this deformation is for now unclear, as vimentin intermediate filaments are not known to form a contractile system, as does acto-myosin. Thus, at this stage, it would appear that this nucleus deformation is the result of the transmission of force generated during the re-arrangement of the vimentin knots. In the later steps of spreading, the vimentin filaments are reorganized into a flat and more homogeneous network. The implications of such strong deformation of the nuclei also remain unclear. It is known that gene expression can be affected by external forces exerted on the nucleus (Miroshnikova et al., 2017). As such, the strong deformation that we have observed here might have also an impact on gene regulation during cell adhesion and spreading.

## Data Availability

All datasets generated for this study are included in the manuscript and/or the Supplementary Files.

## Author Contributions

ET and FL designed the experiments. ET performed the experiments, drafted the figures, and wrote the manuscript. SS helped in the preparation of samples for microscopy. FL revised the manuscript.

## Conflict of Interest Statement

The authors declare that the research was conducted in the absence of any commercial or financial relationships that could be construed as a potential conflict of interest.
