# Supplementary material for: Vimentin Intermediate Filament Rings Deform the Nucleus During the First Steps of Adhesion
Source: Front Cell Dev Biol. 2019 Jun 17;7:106. doi: 10.3389/fcell.2019.00106 (PMC6590062; doi:10.3389/fcell.2019.00106)
Supplement: Supplementary file 5 [file Data_Sheet_1.pdf]

Supplementary Material

1 Supplementary Materials and Methods

Delayed ring formation upon withaferin A treatment

Cells were collected form their culture vessel by trypsinization. They were then centrifuged and re-suspended in medium containing 0.5µM withaferin A or 0.1% DMSO (control). Cells were then placed in an incubator at 37°C with 5% CO2 for 30 min while being kept in suspension in low adhesion slightly open (to allow gas exchange) tubes. After incubation, the cells were again centrifuged and re-suspended in culture medium containing 50 ng/mL Hoechst nuclear staining. 20 000 cells were deposited in a 23 mm glass bottom dish and were allowed to adhere for 1 h in the incubator. The recording of the time-lapse experiment started 1 h 15min after cell deposition. Images were acquired every 5 min.

2 Supplementary Figures

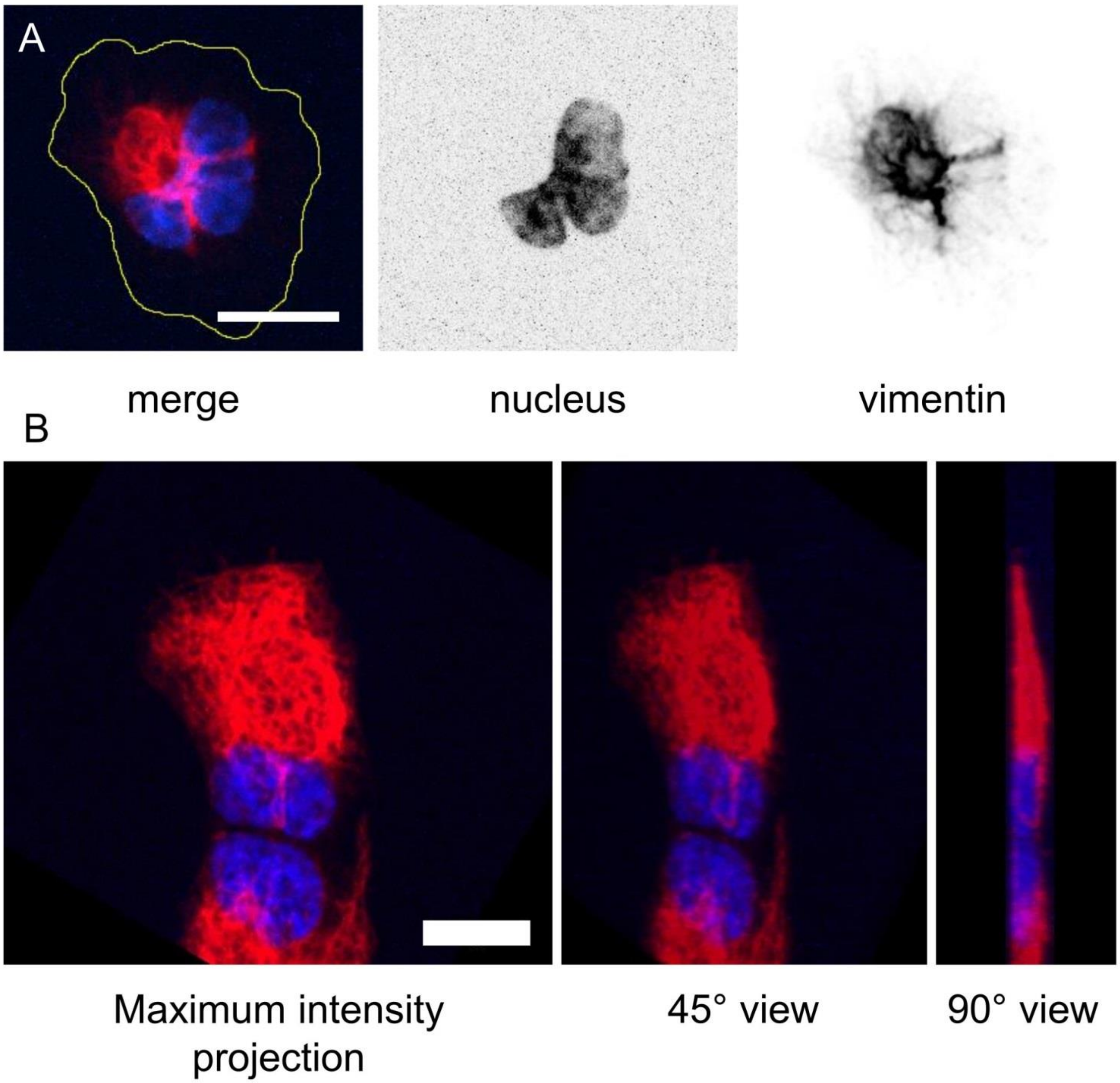

Supplementary Figure 1. Presence of vimentin knots and rings in wild-type RPE1. **(A)** Example of a representative cell bearing a vimentin ring and a deformed nucleus. **(B)** Representative 3D projection of two different neighbor cells. Left: maximum intensity projection; Middle: 45°angle projection; Right: 90° angle projection. Cells were stained after fixing with anti-vimentin antibody and nucleus is counter-stained with Hoechst. Red: Vimentin; Blue: nucleus. Scale bar: 10µm.

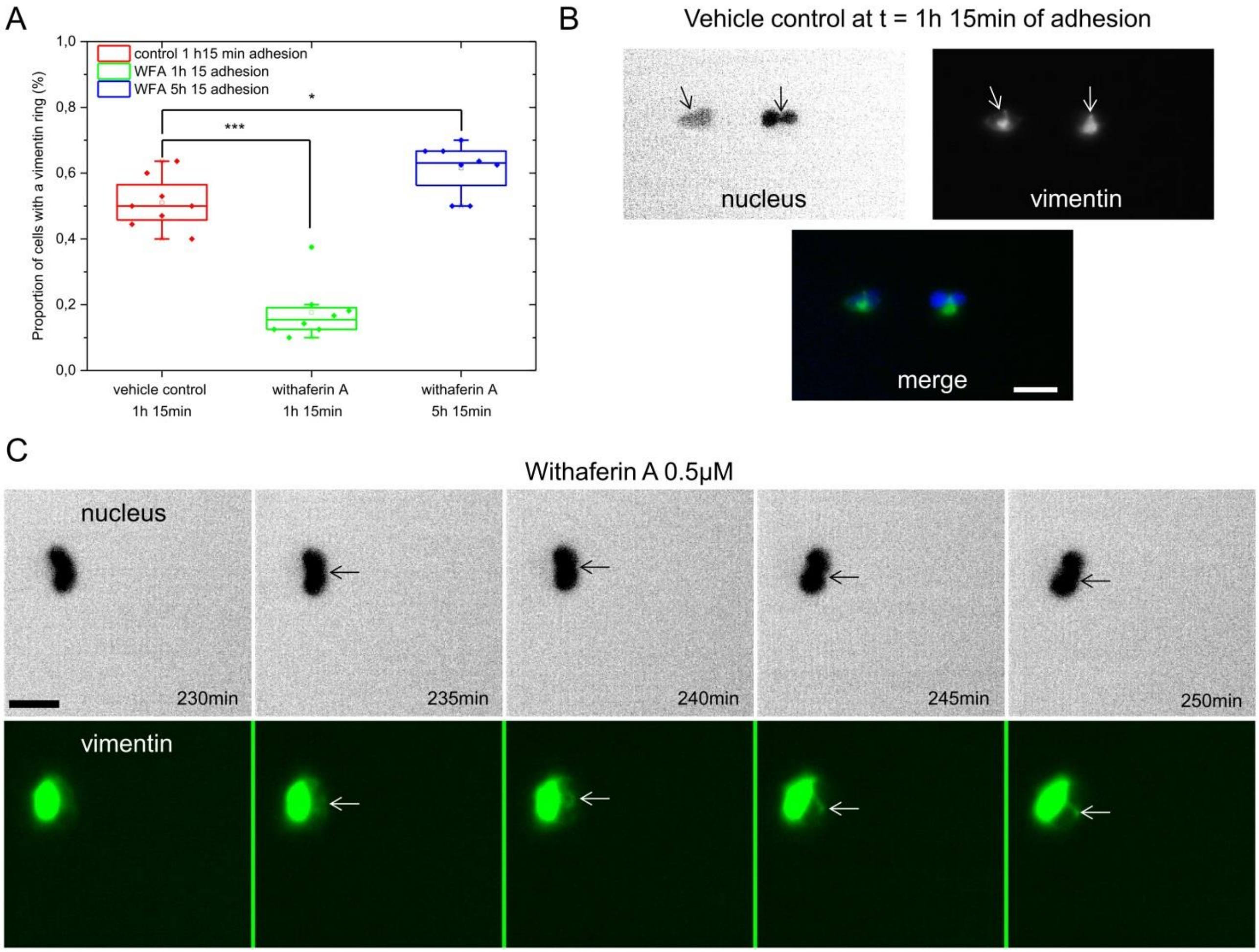

Supplementary Figure 2. Delayed apparition of vimentin rings and nuclear deformations. **(A)** Quantification of the proportion of cells having a vimentin ring at 1h 15 min of adhesion for the vehicle control condition, for the 0.5 µM withaferin A condition, and at 5h 15 min of adhesion for the 0.5µM withaferin A condition.* and ***, $p<0.05$ and $p<0.001$ respectively, Student t-test. Control condition: 96 cells in total; withaferin A condition: 80 cells in total. **(B)** Example of vimentin rings present at 1h 15 min of adhesion (beginning of the recording) for the vehicle control condition. Arrows depict nuclear deformations and are transposed on the vimentin fluorescence images. Green: Vimentin; Blue: nucleus. Scale bar: 20µm. **(C)** Time sequence of the appearance of a vimentin ring within cells incubated 30 minutes with 0.5µM withaferin A before attachment. Arrows depict nuclear deformations and are transposed on the vimentin fluorescence images. Green: Vimentin; Scale bar: 20µm.

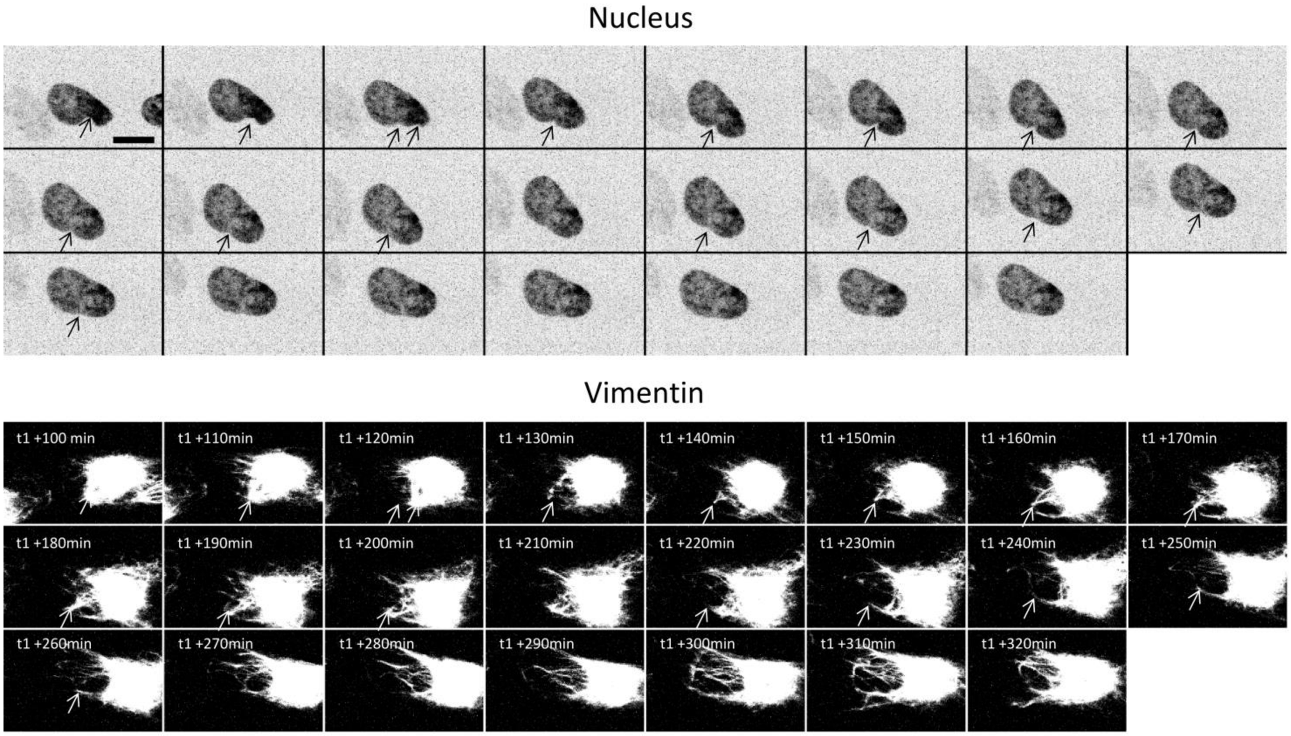

Supplementary Figure 3. Detailed image sequence of Figure 2A with a time period of 10min. Top panel: Nucleus; Bottom panel: Vimentin. Arrows depict nuclear deformations and are transposed on the vimentin fluorescence images. Scale bar: 10µm.

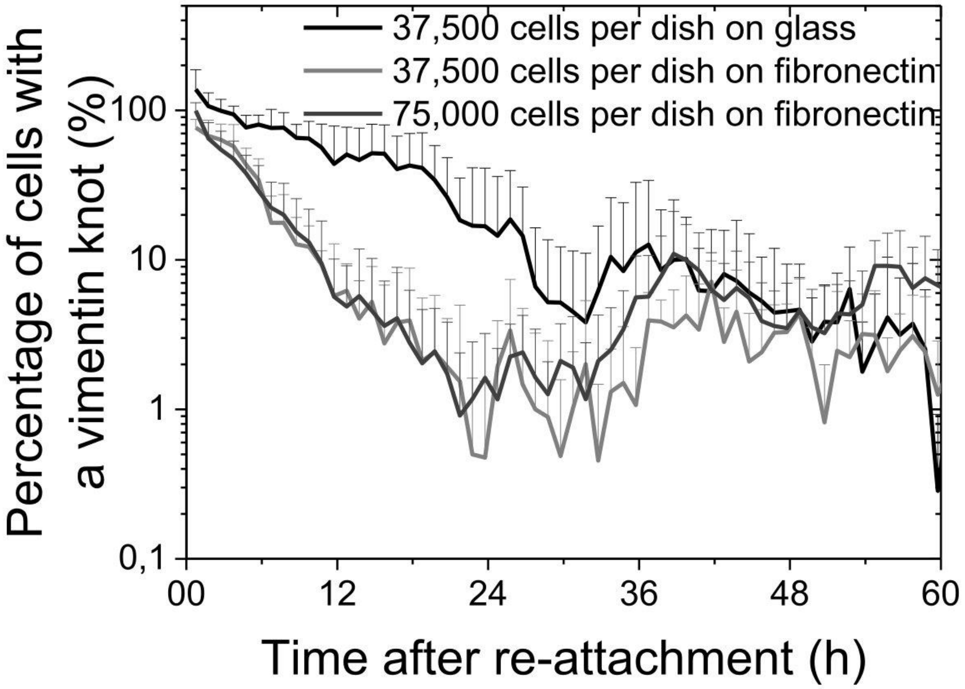

Supplementary Figure 4. Quantification of proportions of HFFs cells with vimentin ball-like structures, over 60 h of cell adhesion and spreading, including different coatings and different cell concentrations. Each time point represents the proportion of knot for a minimum of 125 cells for the 37,500 cells per dish conditions and 250 cells for the 75,00 cells per dish condition.

3 Supplementary movies caption

Movie S1. Control movie of delayed vimentin ring appearance upon withaferin A treatment. Epifluorescence movie acquired with a 20x magnification.

Movie S2. Withaferin A movie of delayed vimentin ring appearance upon withaferin A treatment. Epifluorescence movie acquired with a 20x magnification. Cells were previously incubated with 0.5µM withaferin A for 30 minutes before rinsing.

Movie S3. Sliding of vimentin rings along the nucleus during time. Full time-lapse experiment used in the generation of Figures 2A and S3.

Movie S4. Appearance of vimentin rings during mitosis. Full time-lapse experiment used in the generation of Figure 3.
